# A retrospective study on the impact of SARS-CoV-2 infection on cardiopulmonary function in a healthy Chinese cohort: a focus on “long COVID”

**DOI:** 10.3389/fmed.2026.1916898

**Published:** 2026-08-14

**Authors:** Yingzhe Chen, Rui Li, Zhuhua Zhang

**Affiliations:** Department of Cardiology, Beijing Hospital of Traditional Chinese Medicine, Capital Medical University, Beijing, China

**Keywords:** cardiopulmonary exercise testing, cardiopulmonary fitness, long COVID, overall functional limitations, post-COVID-19 sequelae, retrospective study, SARS-CoV-2

## Abstract

**Objective:**

This retrospective study aimed to observe the cardiopulmonary function and lingering symptoms in previously healthy individuals with Long COVID using Cardiopulmonary Exercise Testing (CPET), the gold standard for evaluating integrated human physiological function.

**Methods:**

A retrospective analysis was conducted on 73 subjects who completed CPET at Beijing Hospital of Traditional Chinese Medicine from January 2023 to April 2023. Participants were consecutively included and comprised 42 patients with confirmed SARS-CoV-2 infection and persistent symptoms (Long COVID group) and 31 healthy controls with no history of COVID-19 (Normal control group). The sample size was determined by the total number of eligible patients who consecutively underwent CPET during the study period, and a post-hoc power analysis confirmed sufficient power (>80%) to detect the observed differences in the primary endpoint (peak $\dot{V}$ O_2_%pred). General clinical data and CPET parameters were retrospectively collected and compared between the two groups. The chi-square test was used for sex distribution, the independent *t*-test IS for normally distributed continuous variables, and the Mann–Whitney *U* test for non-normally distributed variables. Linear regression analysis was performed to explore correlations between peak oxygen uptake (peak $\dot{V}$ O_2_) and other core CPET indicators.

**Results:**

There were 13 males and 18 females in the normal control group and12 males and 30 females in the Long COVID group. No statistical differences were observed in age, sex, height, or weight between the two groups (*p* > 0.05 for all). (2) In the Long COVID group, core CPET indicators were significantly reduced compared to the normal group: peak $\dot{V}$ O2 was78.3 ± 20.7% pred; anaerobic threshold (AT) was 77.9 ± 19.6% pred; peak oxygen pulse (peakO₂pulse) was 100.5 (82.4,110.3)% pred; and Oxygen Uptake Efficiency Plateau (OUEP) was 104.6 ± 12.9% pred (all *p* < 0.05). (3) Other indicators in the Long COVID group also showed significant differences compared to the normal group, including forced vital capacity (FVC) [3.2(2.8,3.8)L], resting heart rate (rest HR) (87.1 ± 9.3 bpm), peak tidal volume (peak VT) [1.4(1.2,1.9)L], Cardiac Power (CP) [3643.8(2868.7,4522.2)], and the slope of oxygen uptake to work rate (ρ $\dot{V}$ O2/ρ WR) [9.6(9.0, 10.54)] (all *p* < 0.05). However, no significant differences were found in maximal voluntary ventilation (MVV) or peak heart rate (peak HR) between the two groups (*p* > 0.05). (4) In the Long COVID group, peak $\dot{V}$ O_2_ showed a significant positive linear correlation with AT (*R*^2^ = 0.63, *p* < 0.001), peakO₂pulse (*R*^2^ = 0.92, *p* < 0.001), and CP (*R*^2^ = 0.44, *p* < 0.001), but not with ρ $\dot{V}$ O2/ρ WR (*R*^2^ = 0.04, *p* = 0.357). (5) Among the Long COVID group, the most frequently reported persistent symptoms were: decreased exercise endurance (19.1%), palpitations (16.1%), chest tightness and shortness of breath (12.6%), fatigue (16.9%), insomnia (7.8%), anxiety (7.3%), and cough (4.8%).

**Conclusion:**

This retrospective analysis confirms that SARS-CoV-2 infection has a significant and not merely short-term impact on the integrated cardiopulmonary and musculoskeletal function. Even after turning negative, individuals exhibit a wide range of symptoms affecting the respiratory, circulatory, and nervous systems. The observed reduction in peak systolic blood pressure, cardiac power, and the ρ $\dot{V}$ O2/ρ WR slope, coupled with compensatory resting tachycardia, suggests the possibility of multiple underlying mechanisms, including subclinical myocardial functional impairment, autonomic nervous system dysfunction, and/or peripheral deconditioning. CPET proved to be a safe and valuable tool for objectively evaluating the overall functional limitations in Long COVID patients.

## 1 Introduction

Since its emergence in 2019, the novel coronavirus (SARS-CoV-2) has caused widespread global infection and millions of deaths. Even though the World Health Organization has declared an end to the COVID-19 public health emergency, the risks associated with SARS-CoV-2 infection persist. A significant concern is that after nucleic acid tests turn negative, many individuals may experience long-term effects across multiple organ systems. This condition is commonly referred to as “Long COVID.” According to the UK National Institute for Health and Care Excellence (NICE), Long COVID encompasses both ongoing symptomatic COVID-19 (symptoms lasting 4 to 12 weeks) and post-COVID-19 syndrome (symptoms persisting beyond 12 weeks) (1). The European Society of Clinical Microbiology and Infectious Diseases (ESCMID) defines it as the presence of one or more symptoms that continue, recur, or resolve beyond 12 weeks after confirmed COVID-19 diagnosis, without an alternative explanation (2). Therefore, SARS-CoV-2 infection is not merely an acute international event; the long-term impact of Long COVID on individual health and public health burdens warrants serious attention and further investigation.

Cardiopulmonary Exercise Testing (CPET) is currently the only non-invasive clinical tool that provides an integrated assessment of overall human functional status. By integrating the theory of cardiopulmonary coupling (CPC) (3) with advanced computer technology, CPET links external respiration, circulation, and internal respiration (cellular metabolism). It provides a scientific, data-driven, and objective quantitative analysis of the coupling between various organs and systems, best reflecting the indivisible nature of the human body as a whole (4). CPET is widely applied in various clinical settings, including preoperative risk assessment for anesthesia, early diagnosis (of myocardial ischemia, heart failure, and pulmonary hypertension), differential diagnosis, objective quantification of disease severity, prognosis prediction for mortality and survival, selection criteria for heart transplantation, monitoring and screening of high-risk populations, guiding exercise prescription, and objectively evaluating treatment efficacy (5–10).

International studies focusing on cardiopulmonary function in Long COVID patients have generally shown a decline in cardiorespiratory fitness as measured by CPET, with a relatively comprehensive analysis across multiple systems (11–14). However, retrospective research in China on the characteristics of CPET core parameters from a holistic perspective is relatively scarce, particularly regarding the overall functional status of patients with “Long COVID” following SARS-CoV-2 infection. Furthermore, while prior studies have characterized exercise limitation in post-COVID states, the novelty of our work lies in providing a detailed, multi-system functional profile using a comprehensive panel of CPET parameters in a previously healthy Chinese cohort, specifically focusing on the interplay between ventilatory, circulatory, and metabolic impairments within a single-center setting (15–17). This may be attributed to the later lifting of pandemic control measures in China, resulting in a lack of scientific data on the overall respiratory, circulatory, and metabolic functions of the Chinese Long COVID population. Therefore, this retrospective study aimed to collect and analyze cardiopulmonary data from the Chinese Long COVID population, thereby laying the foundation for long-term follow-up and subsequent research.

## 2 Methods

### 2.1 Study design and population

This was a single-center retrospective observational study. A total of 73 subjects who completed CPET at Beijing Hospital of Traditional Chinese Medicine from January 2023 to April 2023 were consecutively enrolled. The sample size was determined by consecutive enrollment of all eligible patients during the study period. A post-hoc power analysis using G*Power software (version 3.1.9.7) for an independent t-test (two-tailed, *α* = 0.05) indicated that the achieved sample size (*n* = 42 in Long COVID group, *n* = 31 in control group) provided > 80% power to detect the observed difference in the primary endpoint, peak VO₂ (%pred) (Cohen’s *d* = 1.12). This included 31 healthy controls with no history of COVID-19 (Normal control group) and 42 patients with confirmed SARS-CoV-2 infection and persistent symptoms (Long COVID group). The control group’s CPET data were originally collected as part of routine health check-ups for physically active individuals without any known cardiopulmonary symptoms or diseases. The study was approved by the Ethics Committee of Beijing Hospital of Traditional Chinese Medicine (Ethics number: 2025BL02-086-03) and registered in the Chinese Clinical Trial Registry (ChiCTR2600117659). The requirement for informed consent was waived by the Ethics Committee due to the retrospective nature of the study and the use of anonymized data.

Inclusion criteria:

- *Normal control group*: Individuals with no prior diagnosis of COVID-19 and no abnormal findings on routine health check-ups at our hospital.
- *Long COVID group*: Individuals with a confirmed diagnosis of SARS-CoV-2 infection (positive nucleic acid or antigen test) who met the Long COVID definition criteria proposed by NICE (1) and ESCMID (2), and had no abnormal findings on routine health check-ups.

Exclusion criteria:

- Individuals with pre-existing chronic diseases (e.g., hypertension, diabetes, coronary heart disease, hyperlipidemia, chronic lung disease).
- Patients hospitalized for COVID-19 pneumonia.
- Inability to complete the CPET protocol due to any reason.
- Unconfirmed SARS-CoV-2 infection.

### 2.2 Data collection

Demographic and clinical data were retrospectively extracted from the hospital’s electronic medical records, including age, sex, height, weight, COVID-19 diagnosis date, conversion date, diagnostic method, and persistent symptoms. The duration between the resolution of acute infection (negative nucleic acid test) and CPET performance was recorded. CPET data were retrieved from the hospital’s CPET database. Pre-infection baseline pulmonary function test data were not available for the Long COVID group, as these tests were not part of routine health check-ups for this previously healthy cohort. CPET data were retrieved from the hospital’s CPET database. Symptom data were extracted through medical record review and structured questionnaire collection, covering 12 items across respiratory, cardiovascular, neuropsychological, and systemic symptom domains.

### 2.3 Cardiopulmonary exercise testing (CPET) protocol

All CPETs were performed using a CS-200 Ergospirometry system (Schiller AG, Switzerland), which integrates 12-lead ECG, pulse oximetry, non-invasive blood pressure monitoring, and gas exchange analysis. Daily calibration of the environment, volume, and gas analyzers was performed before testing, according to the manufacturer’s instructions. After signing informed consent, subjects first underwent a seated static pulmonary function test. Subsequently, they performed a symptom-limited incremental exercise test on an electronically braked cycle ergometer, during which 12-lead ECG, oxygen saturation, non-invasive blood pressure, and ventilatory parameters were continuously recorded.

According to the standard continuous incremental ramp protocol from Harbor-UCLA Medical Center (18, 19), the symptom-limited CPET protocol was as follows: 3 min of resting baseline recording, followed by 3 min of unloaded pedaling at 60 rpm. The work rate was then increased continuously using a ramp pattern with an increment of 10–30 W/min, selected based on the subject’s age, sex, disease status, and estimated functional capacity, aiming to reach a symptom-limited maximum within 6–10 min. Subjects were encouraged to reach a Borg score of 19 or above. The test was followed by a recovery period of at least 5 min. The reason for stopping the test was recorded, along with the Borg score. All enrolled participants successfully completed the CPET protocol to symptom-limited maximum exertion. A maximal effort was defined by a respiratory exchange ratio (RER) ≥ 1.10 and/or a Borg score ≥ 18 at peak exercise. No dropouts or premature terminations occurred.

### 2.4 Statistical analysis

Data were managed using Excel with double-entry verification. Statistical analysis was performed using SPSS version 25.0 (IBM Corp., Armonk, NY, United States). The chi-square test was used to compare sex distribution between groups. For continuous variables, normality was assessed using the Shapiro–Wilk test. Normally distributed data with homogeneity of variance were presented as mean ± standard deviation (SD) and compared using independent samples *t*-tests. Non-normally distributed data were presented as median with interquartile range [M (QL, QU)] and compared using Mann–Whitney U tests. A two-tailed *p*-value < 0.05 was considered statistically significant. Linear regression analysis was performed to explore the correlation between peak $\dot{V}$ O_2_ (%pred) and other core CPET indicators, including AT (%pred), peakO₂pulse, △ $\dot{V}$ O_2_/△WR, and CP. Post-hoc power analysis was performed using G*Power software (version 3.1.9.7).

## 3 Results

### 3.1 Comparison of general clinical characteristics

The Long COVID group consisted of 12 males and 30 females, while the normal control group consisted of 13 males and 18 females. There were no statistically significant differences between the two groups in terms of sex distribution (*p* = 0.248, chi-square test), age (*p* = 0.533), height (*p* = 0.953), or weight (*p* = 0.134, Mann–Whitney U test) (Table 1). In the Long COVID group, the median duration between negative nucleic acid conversion and CPET was 86.5 days (IQR: 62.0–115.0 days).

**Table 1:** Comparison of general clinical characteristics between the long COVID group and the normal control group.

Characteristic (unit)	Normal control group (*n* = 31)	Long COVID group (*n* = 42)	*t*/*Z*/*χ*^2^ value	*p* value
Sex (Male/Female)	13/18	12/30	1.333^1^	0.248^1^
Age (years)	43.6 ± 9.9 (26–61)	37.4 ± 8.8 (23–56)	0.627	0.533
Height (cm)	167.0 ± 7.1 (153–178)	167.2 ± 7.3 (153–180)	−0.060	0.953
Weight (kg)	65.0 (56.0, 72.0)	58.0 (51.7, 73.0)	−1.497^2^	0.134^2^

### 3.2 Comparison of Core CPET indicators

As shown in Tables 2, 3, the Long COVID group exhibited significantly lower values for most core CPET indicators compared to the normal control group. Specifically, peak $\dot{V}$ O_2_ (L/min, ml·min^−1^·kg^−1^, %pred), AT (L/min, %pred), peakO₂pulse (ml/beat), and OUEP (ratio, %pred) were all significantly reduced in the Long COVID group (all *p* < 0.05). The differences in peak $\dot{V}$ O_2_ (%pred) and peakO₂pulse (%pred) were particularly pronounced (both *p* < 0.001).

**Table 2:** Comparison of CPET indicators between the Long COVID group and normal control group (*t*-test).

Indicator (unit)	Normal control group (*n* = 31)	Long COVID group (*n* = 42)	*t* value	*P*-value
peak $\dot{V}$ O_2_(%pred)	96.0 ± 8.0 (85–117)	78.3 ± 20.7 (21–132)	5.035	**< 0.001**
peak $\dot{V}$ O_2_ (L/min)	1.9 ± 0.5 (0.4–2.4)	1.5 ± 0.5 (0.29–2.5)	2.817	**0.006**
peak $\dot{V}$ O_2_ (ml·min^−1^·kg^−1^)	29.2 ± 5.8 (20–45)	25.0 ± 6.5 (5–38)	2.797	**0.007**
AT (%pred)	89.8 ± 13.7 (60–121)	77.9 ± 19.6 (39–123)	2.890	**0.005**
AT (L/min)	1.0 ± 0.2 (0.6–1.6)	0.8 ± 0.2 (0.3–1.4)	2.673	**0.009**
peakO₂pulse (ml/beat)	12.3 ± 3.1 (7.9–18.2)	9.9 ± 3.3 (2.4–17.4)	3.093	**0.003**
OUEP (%pred)	115.6 ± 12.6 (93–153)	104.6 ± 13.9 (74–142)	3.447	**0.001**
VE/ $\dot{V}$ CO₂ slope (ratio)	28.1 ± 4.8 (17.7–40.7)	35.0 ± 7.9 (19.1–50.0)	−4.616	**< 0.001**
MVV (L)	134.3 ± 36.9 (80--215.7)	122.6 ± 28.1 (72–195)	1.535	0.129
restHR (bpm)	73.6 ± 9.2 (56–92)	87.1 ± 9.3 (64–110)	−6.081	**< 0.001**
peakHR (bpm)	155.6 ± 13.8 (126–176)	156.9 ± 11.9 (123–181)	−0.421	0.675
peak work load (W)	180.6 ± 50.7 (85–311)	140.7 ± 41.8 (67–272)	3.676	**< 0.001**
peaksysBP (mmHg)	171.1 ± 22.1 (130--210)	144.4 ± 21.6 (105–186)	5.149	**< 0.001**
restRR (breaths/min)	17.6 ± 3.7 (11–27)	19.4 ± 3.9 (10–29)	−2.033	**0.046**
peakRPP (ratio)	26.8 ± 3.7 (18–33)	23.0 ± 3.8 (16–30)	4.163	**< 0.001**
VE/ $\dot{V}$ O_2_-lowest (ratio)	27.2 ± 3.2 (23.0–32.1)	29.3 ± 4.2 (17.4–32.5)	−2.717	**0.008**

Bold values indicate statistically significant correlations (*p* < 0.05) and adjusted *R*^²^ values.

**Table 3:** Comparison of CPET indicators between the long COVID group and normal control group (Mann–Whitney *U* test).

Indicator (unit)	Normal control group (*n* = 31)	Long COVID group (*n* = 42)	*Z* value	*P* value
peakO₂pulse (%pred)	106.1 (101.1, 116.0)	91.6 (77.9, 102.1)	−4.230	**< 0.001**
AT (ml·min^−1^·kg^−1^)	15.5 (14.6, 20.1)	15.0 (13.0, 17.6)	−1.574	0.116
FVC (L)	3.6 (3.0, 4.8)	3.2 (2.8, 3.8)	−2.589	**0.010**
△V˙VO₂/△WR (ratio)	10.3 (9.7, 11.2)	9.6 (9.0, 10.5)	−2.868	**0.004**
rest VT (L)	0.5 (0.4, 0.6)	0.4 (0.4, 0.5)	−2.478	**0.013**
peak VT (L)	1.7 (1.4, 2.1)	1.4 (1.2, 1.9)	−2.003	**0.045**
Cardiac power, CP (ratio)	4700.7 (3958.7, 5978.8)	3643.8 (2868.7, 4522.2)	−4.140	**< 0.001**
peak dia BP (mmHg)	89.0 (80.0, 100.0)	82.5 (74.0, 88.2)	−2.418	**0.016**
OUEP (90s average ratio)	43.2 (40.2, 47.9)	40.0 (35.7, 45.1)	−2.712	**0.007**

Data are presented as median (QL, QU). Bold values indicate statistically significant correlations (*p* < 0.05) and adjusted *R*^²^ values. FVC, forced vital capacity; △$ $\dot{V}$ O_2_/△WR, slope of oxygen uptake to work rate; rest VT, resting tidal volume; peak VT, peak tidal volume; CP, cardiac power; peak dia BP, peak diastolic blood pressure.

### 3.3 Comparison of other CPET indicators

As also shown in Tables 2, 3, several other CPET indicators were significantly different between the two groups. The Long COVID group demonstrated significantly lower values for peak work rate (peakWR), peak systolic blood pressure (peaksysBP), FVC, △ $\dot{V}$ O_2_/△WR, restVT, peakVT, CP, peak diastolic blood pressure (peakdiaBP), and peak rate-pressure product (peakRPP) (all *p* < 0.05). The differences in restHR, peakWR, peaksysBP, peakRPP, and CP were highly significant (all *p* < 0.001). However, no significant differences were observed in MVV or peakHR between the two groups (*p* > 0.05 for both).

### 3.4 Correlation analysis of Core CPET indicators in the long COVID group

**Figure 1:**
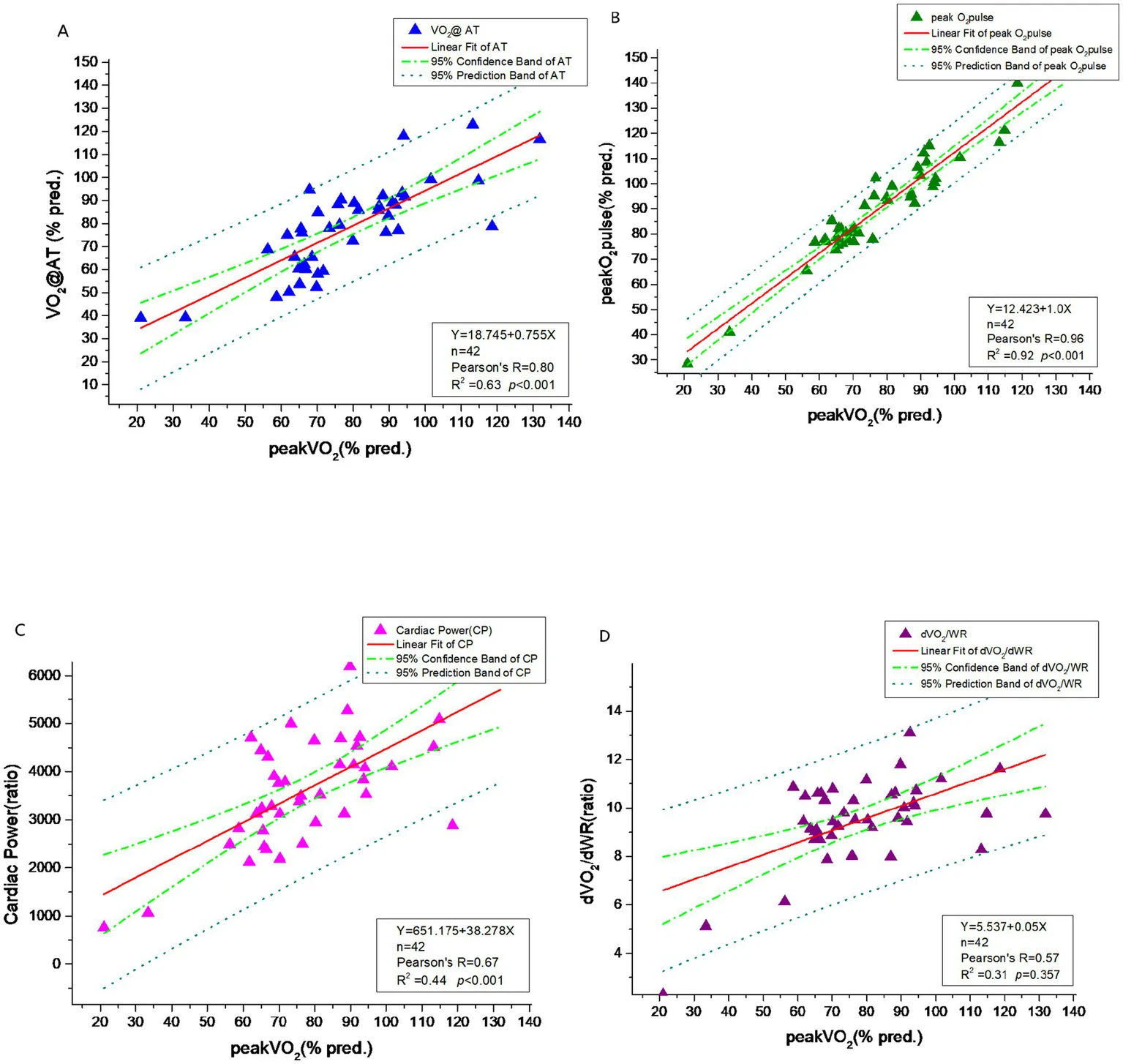
Linear regression analyses between peak oxygen uptake (peak VO₂, %pred) and core CPET parameters in patients with Long COVID (*n* = 42). **(A)** Correlation between peak VO₂ (%pred) and anaerobic threshold (AT, %pred). **(B)** Correlation between peak VO₂ (%pred) and peak oxygen pulse (peak O₂ pulse). **(C)** Correlation between peak VO₂ (%pred) and cardiac power (CP). **(D)** Correlation between peak VO₂ (%pred) and ΔVO₂/WR. Solid red lines represent the linear regression models, and dashed lines indicate the 95% confidence intervals. Pearson correlation coefficients (*R*), coefficients of determination (*R*^2^), and *p* values are shown within each panel.

As presented in Table 4, linear regression analysis revealed that peak $\dot{V}$ O_2_ (%pred) was significantly and positively correlated with AT (%pred), peakO₂pulse, and CP in the Long COVID group. The correlations with AT (%pred) and peakO₂pulse showed particularly strong goodness-of-fit (R^2^ = 0.63 and 0.92, respectively; both *P* < 0.001). However, no significant correlation was found between peak $\dot{V}$ O_2_ (%pred) and △ $\dot{V}$ O_2_/△WR (*R*^2^ = 0.04, *p* = 0.357), The corresponding scatter plots and regression lines are shown in Figure 1.

**Table 4:** Correlation analysis of CPET indicators in the long COVID group.

Dependent variable (Y)	Independent variable (X)	a (intercept)	b (slope)	Pearson’s *R*	*R* ^2^	*P* value
AT (% pred)	peak $\dot{V}$ O_2_ (% pred)	18.745	0.755	0.80	0.63	**< 0.001**
peakO₂pulse (ml/beat)	peak $\dot{V}$ O_2_(% pred)	12.423	1.000	0.96	0.92	**< 0.001**
CP (ratio)	peak $\dot{V}$ O_2_(% pred)	651.17	28.278	0.67	0.44	**< 0.001**
△V˙VO₂/△W*R* (ratio)	peak $\dot{V}$ O_2_ (% pred)	5.537	0.050	0.20	0.04	0.357

Y = a + bX, where Y represents the predicted percentage value of various CPET core indicators, and X represents the predicted percentage value of peak oxygen uptake. Bold values indicate statistically significant correlations (*p* < 0.05) and adjusted *R*^²^ values. CP, cardiac power; △ $\dot{V}$ O_2_/△WR, slope of oxygen uptake to work rate.

### 3.5 Analysis of persistent symptoms in the long COVID group

**Figure 2:**
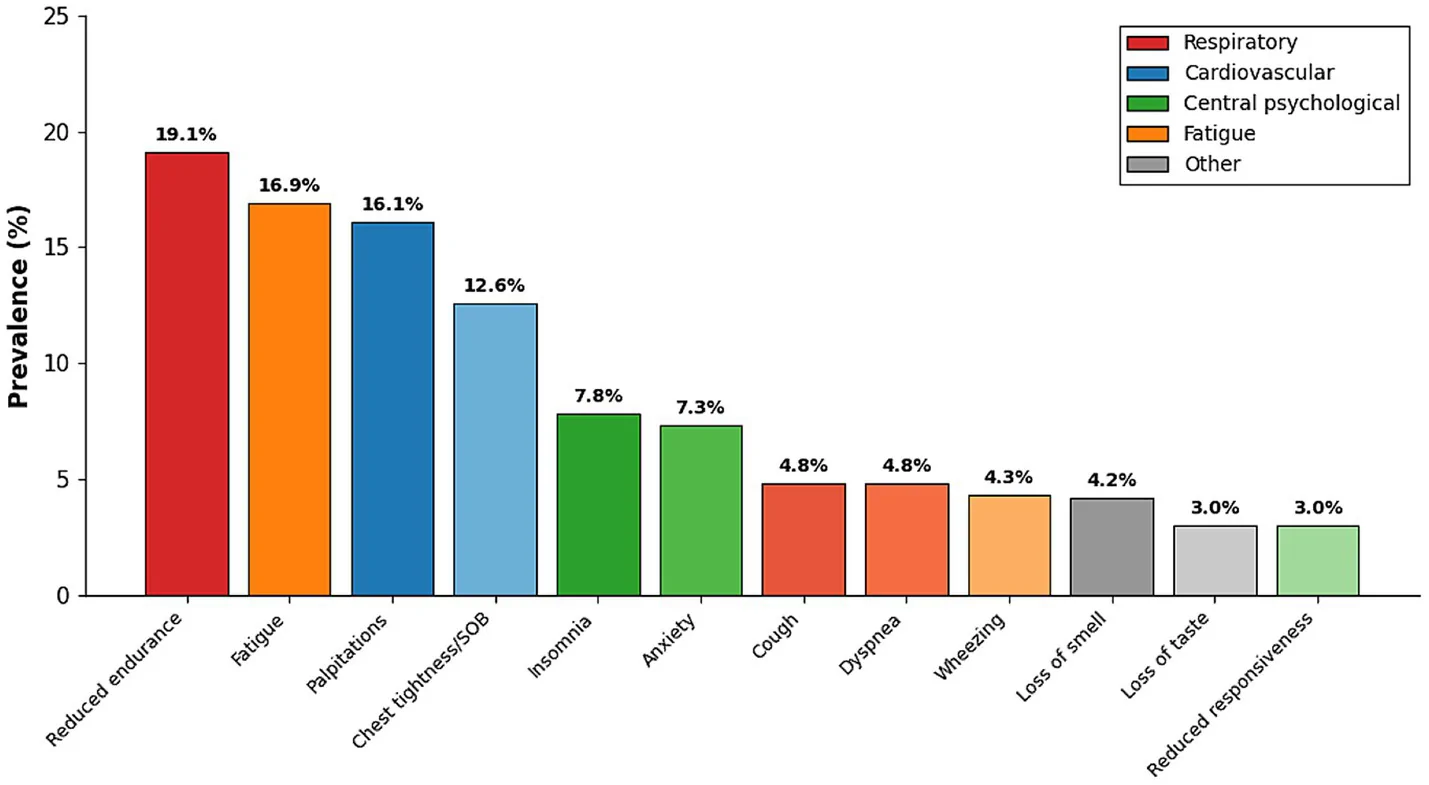
Prevalence of persistent symptoms in long COVID group (*n* = 42). Symptoms are categorized into five domains: respiratory (dyspnea, cough, wheezing, and decreased exercise endurance), cardiovascular (chest tightness/shortness of breath and palpitations), central psychological (insomnia, decreased responsiveness, and anxiety), fatigue, and other (loss of smell and loss of taste). The three most frequently reported symptoms were decreased exercise endurance (19.1%), fatigue (16.9%), and palpitations (16.1%).

Based on retrospective review of medical records, the most frequently reported persistent symptoms among the 42 patients in the Long COVID group were categorized as follows (Figure 1):

- *Respiratory system*: dyspnea (4.8%, 2/42), cough (4.8%, 2/42), wheezing (4.3%, 2/42), and decreased exercise endurance (19.1%, 32/42).
- *Cardiovascular system*: chest tightness and shortness of breath (12.6%, 21/42), palpitations (16.1%, 27/42).
- *Central psychological symptoms*: insomnia (7.8%, 13/42), decreased responsiveness (3.0%, 5/42), anxiety (7.3%, 12/42).
- *Fatigue*: 16.9% (28/42).
- *Other*: decreased or loss of smell (4.2%, 7/42), decreased or loss of taste (3.0%, 5/42) Figure 2.

## 4 Discussion

### 4.1 Core indicators of overall functional status in CPET

This retrospective study demonstrates that in the Long COVID group, core CPET indicators—including peak $\dot{V}$ O_2_ (%pred), AT (%pred), peakO₂pulse (%pred), △ $\dot{V}$ O_2_/△WR, and CP—were all significantly lower than those in the normal control group. Furthermore, peak $\dot{V}$ O_2_ showed a significant positive linear correlation with AT, peakO₂pulse, and CP, indicating that these parameters effectively reflect the integrated pathophysiological status of cardiopulmonary-metabolic coupling, gas exchange, and hemodynamics in Long COVID patients.

Peak oxygen uptake (peak $\dot{V}$ O_2_) and peak oxygen pulse (peakO₂pulse) are key indicators of the coupling and matching between the respiratory, circulatory, and peripheral skeletal muscle metabolic systems (5). The anaerobic threshold (AT) represents the inflection point during incremental exercise at which the mitochondrial energy supply shifts from predominantly aerobic to anaerobic metabolism. It is the highest oxygen consumption value at which blood lactate concentration and the lactate/pyruvate ratio do not continuously increase during prolonged submaximal exercise, reflecting the body’s oxygen transport capacity (4). Cardiac power (CP) and the △ $\dot{V}$ O_2_/△WR slope reflect the ability of muscles to extract oxygen. The reduction in these parameters suggests an imbalance in muscle oxygen supply and demand, primarily due to circulatory impairment. The concurrent reduction in AT further supports this interpretation and aids in the diagnosis of cardiovascular dysfunction.

Our findings align with several recent studies that have reported impaired exercise capacity and oxygen extraction in Long COVID patients (15–17). For instance, Norweg et al. (15) demonstrated that exercise intolerance in Long COVID was associated with impaired peripheral oxygen extraction, while Aparisi et al. (17) highlighted the value of CPET in identifying exercise limitation in post-acute sequelae of COVID-19. Our study extends these observations by providing a comprehensive profile of multiple CPET parameters in a previously healthy Chinese cohort.

From a pathophysiological perspective, SARS-CoV-2 infection affects multiple systems (20). In the respiratory system, the virus directly invades lung tissue, indirectly triggers inflammatory responses, or causes endothelial injury that activates fibroblasts, ultimately leading to pulmonary fibrosis. In the cardiovascular system, the high expression of ACE2 receptors in the heart allows direct viral entry and indirect inflammatory activation, resulting in myocyte injury and death. Additionally, virus-induced afferent autonomic nervous system dysfunction can lead to complications such as tachycardia syndrome. Regarding central psychological symptoms, prolonged immune responses activate glial cells and damage neurons; viral effects on the brain contribute to cognitive impairment; and chronic brainstem inflammation leads to autonomic dysfunction (20). Common fatigue is thought to be related to nerve injury caused by systemic inflammation and cell-mediated immune mechanisms, as well as muscle fiber damage or atrophy (21).

### 4.2 Cardiovascular response during CPET in the long COVID population

Beyond the core indicators, cardiovascular responses during CPET—including heart rate, blood pressure, cardiac power index, △ $\dot{V}$ O_2_/△WR, and myocardial oxygen consumption index—provide valuable insights into functional abnormalities. In this retrospective analysis, the Long COVID group exhibited resting tachycardia, along with significantly lower peak systolic and diastolic blood pressure, cardiac power, △ $\dot{V}$ O_2_/△WR, and myocardial oxygen consumption index compared to the normal group.

The observed reduction in peak systolic blood pressure, cardiac power, and the △ $\dot{V}$ O_2_/△WR slope, coupled with compensatory resting tachycardia, suggests the possibility of multiple underlying mechanisms. While these findings could indicate subclinical myocardial functional impairment, they could also be explained by autonomic nervous system dysfunction [e.g., Postural Orthostatic Tachycardia Syndrome (POTS)], peripheral microvascular/endothelial dysfunction, or physical deconditioning. Some international studies attribute post-COVID tachycardia to autonomic nervous system dysfunction (22, 23). From a functional perspective, our findings do not exclude autonomic involvement but also raise the possibility of subtle myocardial dysfunction. However, in the absence of confirmatory cardiac imaging (e.g., echocardiography, cardiac MRI) or specific biomarkers (e.g., high-sensitivity troponin, NT-proBNP), we cannot definitively attribute these findings to myocardial damage alone. This retrospective finding warrants further investigation, including correlation with cardiac imaging or more sensitive biomarkers. The lack of such data is a significant limitation of our study, which we have acknowledged in the limitations section.

### 4.3 Impact on pulmonary function in the long COVID population

SARS-CoV-2 primarily enters the body via the respiratory tract, making the respiratory system one of the first to be affected. Common symptoms such as cough, sputum production, and sore throat are frequently reported. In this retrospective study, CPET revealed that the Long COVID group had significantly lower FVC, rest VT, and peak VT, along with a significantly higher rest RR compared to normal controls. These findings indicate that viral infection led to compensatory tachypnea due to reduced tidal volume and vital capacity. Therefore, the impact of SARS-CoV-2 on pulmonary function in Long COVID patients is evident, consistent with findings from both domestic and international studies (24–26). However, the absence of pre-infection baseline pulmonary function tests limits our ability to definitively rule out pre-existing, subclinical pulmonary abnormalities or to differentiate between parenchymal lung disease, pulmonary vascular disease, or respiratory muscle weakness as primary drivers of the observed ventilatory inefficiency. This is an important caveat to our findings.

### 4.4 Clinical symptom manifestations in the long COVID population

The persistent symptoms in Long COVID patients are characterized by their wide range, multiplicity, and long duration, and can even occur after breakthrough infections in vaccinated individuals (27–29). The five major symptom categories identified retrospectively in this study—respiratory (dyspnea, cough, wheezing, decreased exercise endurance), cardiovascular (chest tightness, shortness of breath, palpitations), central psychological (insomnia, decreased responsiveness, anxiety), fatigue (weakness), and other (loss of smell/taste)—underscore that the impact of SARS-CoV-2 extends far beyond a single organ system, involving multiple systems throughout the body. We have corrected the previously reported dyspnea rate from 0.51 to 4.8% based on a re-review of the medical records, which aligns more consistently with the symptom burden in this cohort.

### 4.5 Safety of CPET in the long COVID population

As a tool for assessing overall functional status, CPET played a crucial role in this study, providing functional insights into the respiratory, circulatory, and metabolic status of Long COVID patients after infection. Importantly, no adverse clinical events (e.g., severe dyspnea, sudden cardiac death, malignant hypertension) occurred during any CPET procedure. Follow-up after testing revealed no worsening of symptoms or other adverse effects. Therefore, CPET is confirmed as a safe and effective tool for objective, comprehensive functional assessment in Long COVID patients and can serve as the best objective, quantitative method for long-term follow-up.

## 5 Conclusion

The impact of Long COVID on cardiorespiratory fitness is a critical topic. Accumulating evidence suggests that patients may experience various health effects, including those impacting cardiopulmonary health. This retrospective analysis demonstrates that individuals with Long COVID exhibit a significant reduction in overall functional status, affecting not a single organ but rather an integrated network involving the respiratory, cardiovascular, and musculoskeletal systems. These findings align with other research (17, 30). As infection rates continue and reinfections become more common, the cumulative risk of post-acute sequelae may increase. Thus, objective, holistic functional assessment is essential.

This retrospective study suggests the possibility of subclinical myocardial functional impairment in previously healthy individual’s post-COVID-19, as evidenced by reduced peak systolic blood pressure, cardiac power, and the △ $\dot{V}$ O_2_/△WR slope, along with compensatory resting tachycardia. However, autonomic dysfunction and deconditioning remain important alternative explanations. This finding warrants long-term follow-up beyond serological monitoring, ideally incorporating cardiac imaging and biomarker assessment. For Long COVID patients, regular cardiopulmonary functional assessments and individualized rehabilitation programs are crucial to aid recovery and enhance cardiopulmonary health and endurance.

## 6 Limitations

This study has several limitations inherent to its retrospective design. First, it is a single-center study with a relatively small sample size, which may limit the generalizability of the findings. Although post-hoc power analysis confirmed sufficient power for the primary endpoint, the sample size was not predetermined by a formal prospective power calculation. Second, the retrospective nature precludes the establishment of causality; only associations can be inferred. Third, serological markers of myocardial injury (e.g., troponin, CK-MB) and cardiac imaging data (e.g., echocardiography, cardiac MRI) were not systematically collected, severely limiting our ability to correlate functional findings with structural or biochemical evidence of cardiac damage and to definitively attribute the observed cardiovascular limitations to myocardial pathology versus other etiologies such as autonomic dysfunction or deconditioning. Fourth, the lack of long-term follow-up data prevents assessment of the persistence or resolution of the observed functional impairments. Fifth, selection bias may exist as all participants were from a single tertiary hospital. Sixth, the absence of pre-infection baseline pulmonary function tests limits our ability to rule out pre-existing pulmonary conditions as contributors to the observed ventilatory inefficiency. Future prospective, multicenter studies with larger sample sizes, longer follow-up periods, and comprehensive cardiac and pulmonary imaging/biomarker panels are needed to validate these findings and better understand the duration and extent of SARS-CoV-2’s impact on human physiology.

## Data Availability

The raw data supporting the conclusions of this article will be made available by the authors, without undue reservation.

## References

[1] National Institute for Health and Care Excellence (NICE). COVID-19 Rapid Guideline: Managing the Long-Term Effects of COVID-19. London: NICE (2022). 33555768

[2] Yelin D Moschopoulos CD Margalit I Gkrania-Klotsas E Landi F Stahl JP . ESCMID rapid guidelines for assessment and management of long COVID. Clin Microbiol Infect. (2022) 28:1049–59. doi: 10.1016/j.cmi.2022.04.004 PMC8849856 35182760

[3] Whipp BJ Ward SA. Cardiopulmonary coupling during exercise. J Exp Biol. (1982) 100:175–93. doi: 10.1242/jeb.100.1.175, 6816892

[4] Wasserman K Hansen JE Sue DY Stringer W. Principles of Exercise Testing and Interpretation: Including Pathophysiology and Clinical Applications. Philadelphia: Lippincott Williams and Wilkins (2012).

[5] Sun XG Hansen JE Stringer WW. Oxygen uptake efficiency plateau best predicts early death in heart failure. Chest. (2012) 141:1284–94. doi: 10.1378/chest.11-2042, 22030802 PMC4694089

[6] Mehra MR Canter CE Hannan MM Semigran MJ Uber PA Baran DA . The 2016 International Society for Heart Lung Transplantation listing criteria for heart transplantation: a 10-year update. J Heart Lung Transplant. (2016) 35:1–23. doi: 10.1016/j.healun.2015.10.019 26776864

[7] Guazzi M Arena R Halle M Piepoli MF Myers J Lavie CJ. 2016 focused update: clinical recommendations for cardiopulmonary exercise testing data assessment in specific patient populations. Circulation. (2016) 133:e694–711. doi: 10.1161/CIR.0000000000000406 27143685

[8] Chen YZ Sun XG Zhang Y. From cardiopulmonary resuscitation, cardiopulmonary exercise test and cardiopulmonary coupling to new theory of holistic integrative physiology and medicine. J Hum Physiol Rev Article. (2020) 3:1–16. doi: 10.31829/2691-5391/jhp2020-3(1)-106

[9] Magrì D Corrà U Di Lenarda A Cattadori G Maruotti A Iorio A . Cardiovascular mortality and chronotropic incompetence in systolic heart failure: the importance of a reappraisal of current cut-off criteria. Eur J Heart Fail. (2014) 16:201–9. doi: 10.1002/ejhf.24, 24464973

[10] Chen YZ Sun XG Tai WQ Song Y Shi C Hao L . The characteristics of core parameters during cardiopulmonary exercise testing in patients with hypertrophy cardiomyopathy. Chin. J. Appl. Physiol. (2021) 37:65–571. doi: 10.12047/j.cjap.0095.2021.107 34672465

[11] Barbagelata L Masson W Iglesias D Lillo E Migone JF Orazi ML . Cardiopulmonary exercise testing in patients with post-COVID-19 syndrome. Med Clin (Barc). (2022) 159:6–11. doi: 10.1016/j.medcli.2021.07.022 PMC8318690 34417020

[12] Clavario P De Marzo V Lotti R Barbara C Porcile A Russo C . Cardiopulmonary exercise testing in COVID-19 patients at 3 months follow-up. Int J Cardiol. (2021) 340:113–8. doi: 10.1016/j.ijcard.2021.08.027, 34311011 PMC8302817

[13] Rinaldo RF Mondoni M Parazzini EM Pitari F Brambilla E Luraschi S . Deconditioning as main mechanism of impaired exercise response in COVID-19 survivors. Eur Respir J. (2021) 58:2100870. doi: 10.1183/13993003.00870-2021, 33926969 PMC8082950

[14] Schwendinger F Knaier R Radtke T Schmidt-Trucksäss A. Low cardiorespiratory fitness post-COVID-19: a narrative review. Sports Med. (2023) 53:51–74. doi: 10.1007/s40279-022-01751-7, 36115933 PMC9483283

[15] Norweg A Yao L Barbuto S Nordvig AS Tarpey T Collins E . Exercise intolerance associated with impaired oxygen extraction in patients with long COVID. Respir Physiol Neurobiol. (2023) 313:104062. doi: 10.1016/j.resp.2024.104362 37076024 PMC10108551

[16] Barbagelata L Masson W Iglesias D Lillo E Migone JF Orazi ML . Cardiopulmonary Exercise Testing in Patients with Post-COVID-19 Syndrome. *Med Clin (Barc)*. (2022) 159:6–11. doi: 10.1016/j.medcli.2021.07.007 PMC8318690 34417020

[17] Aparisi A Ladrón R Ybarra-Falcón C Tobar J San Román JA. Exercise intolerance in post-acute sequelae of COVID-19 and the value of cardiopulmonary exercise testing- a mini-review. Front Med (Lausanne). (2022) 9:924819. doi: 10.4103/ecdt.ecdt_43_24 35935782 PMC9352932

[18] Sun XG Hansen JE Stringer WW. The new 9 panels display of data from cardiopulmonary exercise test, emphasizing holistic integrative multi-systemic functions. Chin J Appl Physiol. (2015) 31:369–73. 26775513

[19] Sun XG. Standardizing clinical performance, data analysis, graphics display, interpretation and report for cardiopulmonary exercise testing. Chin J Appl Physiol. (2015) 31:361–5. 26775511

[20] Crook H Raza S Nowell J Young M Edison P. Long COVID—mechanisms, risk factors, and management. BMJ. (2021) 374:n1648. doi: 10.1136/bmj.n1648, 34312178

[21] Xie L Zhang Z Wang Q Chen Y Lu D Wu W. COVID-19 and diabetes: a comprehensive review of angiotensin converting enzyme 2, mutual effects and pharmacotherapy. Front Endocrinol (Lausanne). (2021) 12:772865. doi: 10.3389/fendo.2021.772865, 34867819 PMC8639866

[22] Inanc IH Sabanoglu C. Autonomic dysfunction and metabolic disorders as the possible sequelae of COVID-19 infection. Eur Rev Med Pharmacol Sci. (2022) 26:5587–95. doi: 10.26355/eurrev_202208_29444, 35993657

[23] Menezes Junior ADS Schröder AA Botelho SM Resende AL. Cardiac Autonomic Function in Long COVID-19 Using Heart Rate Variability: An Observational Cross-Sectional Study. J Clin Med. (2022) 12:100. doi: 10.3390/jcm12010100 36614901 PMC9821736

[24] Thomas M Price OJ Hull JH. Pulmonary function and COVID-19. Curr Opin Physio. (2021) 21:29–35. doi: 10.1016/j.cophys.2021.03.002, 33817453 PMC7997144

[25] Fresard I Genecand L Altarelli M Gex G Vremaroiu P Vremaroiu-Coman A . Dysfunctional breathing diagnosed by cardiopulmonary exercise testing in 'long COVID' patients with persistent dyspnoea. BMJ Open Respir Res. (2022) 9:e001222. doi: 10.1136/bmjresp-2022-001222 PMC8968537 35354589

[26] Besnier F Bérubé B Malo J Gagnon C Grégoire CA Juneau M . Cardiopulmonary rehabilitation in long-COVID-19 patients with persistent breathlessness and fatigue: the COVID-rehab study. Int J Environ Res Public Health. (2022) 19:4133. doi: 10.3390/ijerph19074133, 35409815 PMC8998214

[27] Bull-Otterson L Baca S Saydah S Boehmer TK Adjei S Gray S . Post-COVID conditions among adult COVID-19 survivors aged 18-64 and ≥65 years—United States, march 2020—November 2021. MMWR Morb Mortal Wkly Rep. (2022) 71:713–7. doi: 10.15585/mmwr.mm7121a1

[28] Al-Aly Z Bowe B Xie Y. Long COVID after breakthrough SARS-CoV-2 infection. Nat Med. (2022) 28:1461–7. doi: 10.1038/s41591-022-01840-0, 35614233 PMC9307472

[29] Naeije R Caravita S. Phenotyping long COVID. Eur Respir J. (2021) 58:2101240. doi: 10.1183/13993003.01240-2021 PMC8287735 34244323

[30] Norweg A Yao L Barbuto S Nordvig AS Tarpey T Collins E . Exercise intolerance associated with impaired oxygen extraction in patients with long COVID. Respir Physiol Neurobiol. (2023) 313:104062. doi: 10.1016/j.resp.2023.104062, 37076024 PMC10108551

